# Carbon Dots: A New Carbon Nanomaterials for Catalyzing Water Electrolysis

**DOI:** 10.1021/polymscitech.5c00001

**Published:** 2025-01-24

**Authors:** Han Wu, Siyu Lu

**Affiliations:** † College of Chemistry and Pingyuan Laboratory, 12636 Zhengzhou University, Zhengzhou 450001, P.R. China

The development of hydrogen energy is essential for global decarbonization.^1,2^ In recent years, carbon nanomaterials with novel structures, multiple components, and functions have garnered significant attention for electrocatalytic water splitting to produce hydrogen. Carbon dots (CDs), characterized by particle sizes smaller than 10 nm, are considered quasi-zero-dimensional carbon nanomaterials. CDs feature an sp^2^-/sp^3^-hybridized carbon core with surfaces enriched by functional groups, such as carboxy, amine, and hydroxyl. The small size and compositional flexibility of CDs impart them with exceptional properties, including tunable electronic structures, good dispersibility, adjustable photoluminescence, and unique electron donor–acceptor characteristics. These attributes make CDs highly effective for applications in photo-/electrocatalysis and energy storage. Furthermore, the abundance of precursors, such as biomass and organic small molecules, and simple preparation processes position CDs as ideal carbon nanomaterials.

Recent research by our group has focused on designing and synthesizing novel CDs and CD-based nanocomposites with precise atomic-level control. We proposed a “hybrid carbon core and polymer shell” structure model of CDs, as shown in Figure 1. Additionally, we developed a “CDs-confined metal-based nanocrystalline” strategy to produce high-performance CD-based nanomaterials for water electrolysis. Chang et al. utilized a facile hydrothermal method to synthesize highly crystalline CDs.^3^ These graphitized CDs, with an average diameter of 6.0 nm, offer abundant adsorption sites for anchoring metal species. When used as support for Fe_3_C nanocrystals and Fe single atoms, the CDs significantly enhanced the catalyst’s performance of oxygen reduction and oxygen evolution (Figure 2a). Moreover, the cross-linking between CDs and metal ions can form 3D network structures, which can effectively enhance charge transfer and material transport. Song et al. developed a halogen-doped CDs-modified amorphous cobalt phosphide (X-CDs/CoP, X representing F, Cl, or Br) as both cathode and anode electrocatalysts for water electrolysis (Figure 2b).^4^ The introduction of CDs effectively adjusted the geometric structure and composition of the X-CDs/CoP catalysts. The unique 3D network structure also provided abundant active sites and ensured fast charge transfer. The synthesized F-CDs/CoP/NF catalyst exhibited good activity and excellent stability (Figure 2c,d).

**Figure 1:**
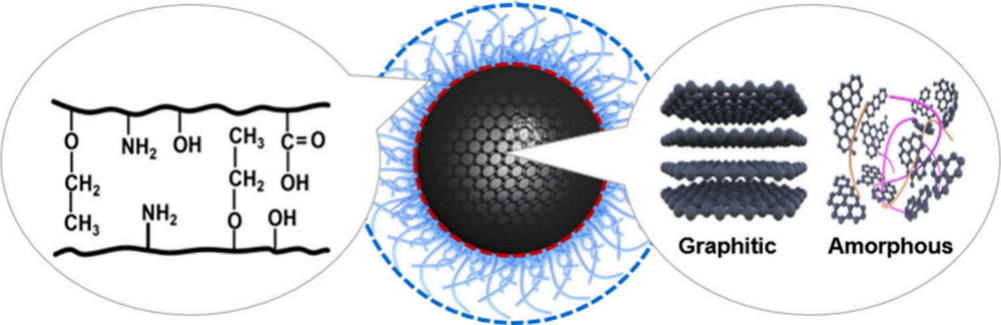
A “hybrid carbon core and polymer shell” structure model of CDs.

**Figure 2:**
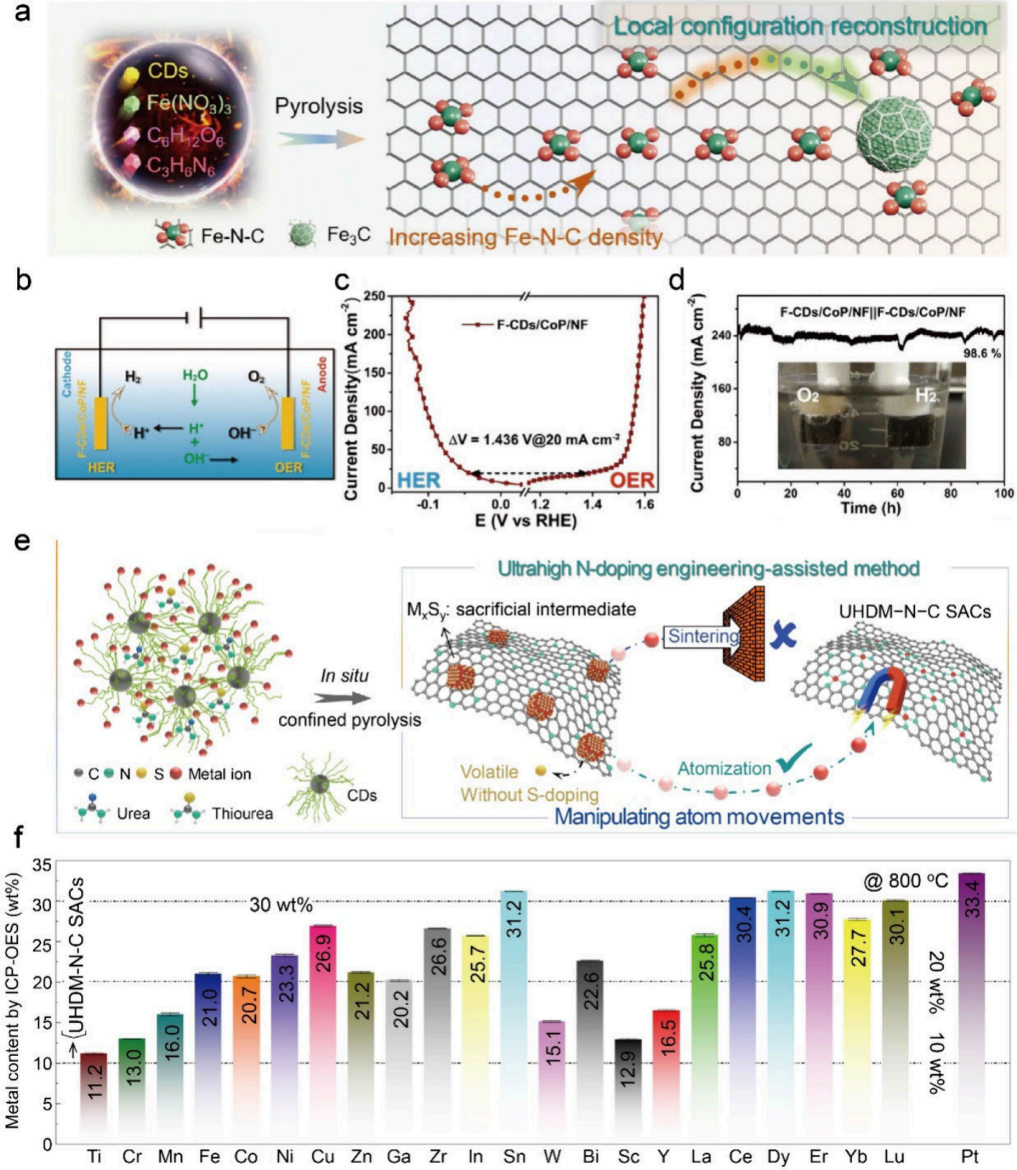
CD-based nanocomposites for catalyzing water electrolysis. (a) Schematic illustration of CD-assisted synthesis of Fe–N–C/Fe_3_C composites. Reproduced from ref 3. Available under a CC BY license. Copyright 2023 The Authors. (b–d) Performance of water electrolysis using F-CDs/CoP/NF as both cathode and anode. Reproduced with permission from ref 4. Copyright 2022 Wiley-VCH. (e, f) 23 kinds of ultra-high-density metal single-atom catalysts using CDs as building blocks. Reproduced from ref 5. Available under a CC BY license. Copyright 2024 Nature Publishing Group.

Due to their large specific surface area, excellent dispersion, and strong coordination interactions with metal ions, CDs promise to develop high-performance metal single-atom catalysts. Chang et al. proposed a “metal sulfide-mediated atomization process” to construct ultra-high-density metal single-atom catalysts using highly graphitized CDs as the foundation (Figure 2e,f).^5^ This strategy successfully synthesized 23 types of metal single-atom catalysts with a metal loading capacity of more than 10.0 wt %. Among them, Ni single-atom catalysts demonstrated outstanding oxygen evolution performance. Notably, these catalysts were scaled up successfully under experimental conditions.

## Conclusions and Outlook

Despite significant progress in developing CDs and CD-based nanomaterials, several challenges remain. Current detection technologies do not provide direct observations of the growth processes of CDs, leaving their growth mechanisms, structures, and compositions insufficiently understood. Innovative synthetic strategies and design concepts are needed to accelerate the application of CDs in energy storage and conversion. Furthermore, the specific roles of CDs in catalytic reactions should be elucidated using advanced *in situ* detection techniques, such as X-ray absorption spectroscopy, X-ray photoelectron spectroscopy, and scanning transmission electron microscopy. These methods establish correlations between the structures and activities of CD-based electrocatalysts, providing deeper insights into their functionalities.
